# Developing an e-learning course on the use of PRO measures in oncological practice: health care professionals’ preferences for learning content and methods

**DOI:** 10.1007/s00520-021-06676-x

**Published:** 2021-11-19

**Authors:** Monika Sztankay, Lisa M. Wintner, Sigrid Roggendorf, Thomas Nordhausen, Linda Dirven, Martin J. B. Taphoorn, Irma M. Verdonck-de Leeuw, Galina Velikova, Andrew Bottomley, Dagmara Kulis, Timo Kachel, Heike Schmidt, on behalf of the EORTC Quality of Life Group

**Affiliations:** 1 University Hospital of Psychiatry II, Medical University of Innsbruck, Anichstrasse 35, Innsbruck, Austria; 2 University Hospital of Psychiatry I, Medical University of Innsbruck, Innsbruck, Austria; 3 Institute for Health and Nursing Science, Medical Faculty, Martin Luther University Halle-Wittenberg, Halle, Germany; 4 Department of Neurology, Leiden University Medical Center, Leiden, The Netherlands; 5 Department of Neurology, Haaglanden Medical Center, The Hague, The Netherlands; 6 Department of Otolaryngology-Head and Neck Surgery, Amsterdam University Medical Centers (Location VUmc), Amsterdam UMC, Vrije Universiteit Amsterdam, Cancer Center Amsterdam, Amsterdam, The Netherlands; 7 Faculty of Behavioural and Movement Sciences, Section Clinical Psychology, Amsterdam Public Health, Amsterdam, The Netherlands; 8 Leeds Institute of Medical Research at St James’s, University of Leeds, Leeds, UK; 9 Leeds Cancer Centre, St James’s University Hospital, Leeds, UK; 10 European Organisation for Research and Treatment of Cancer (EORTC), Quality of Life Department, Brussels, Belgium; 11 Department for Radiation Medicine, University Hospital Halle (Saale), Halle, Germany

**Keywords:** Patient-reported outcome measures, Oncology, Quality of life, EORTC, e-learning, Distant learning, Implementation

## Abstract

**Purpose:**

Implementation of patient-reported outcome measures (PROMs) in clinical routine requires knowledge and competences regarding their use. In order to facilitate implementation, an e-learning course for health care professionals (HCPs) on the utilisation of European Organisation for Research and Treatment of Cancer (EORTC) PROMs in oncological clinical practice is being developed. This study aimed to explore future users’ educational needs regarding content and learning methods.

**Methods:**

The sequential mixed methods approach was applied. A scoping literature review informed the guideline for qualitative interviews with HCPs with diverse professional backgrounds in oncology and cancer advocates recruited using a purposive sampling strategy. An international online survey was conducted to validate the qualitative findings.

**Results:**

Between December 2019 and May 2020, 73 interviews were conducted in 9 countries resulting in 8 topic areas (Basic information on PROs in clinical routine, Benefits of PRO assessments in clinical practice, Implementation of PRO assessments in clinical routine, Setup of PRO assessments for clinical application, Interpretation of PRO data, Integration of PROs into the communication with patients, Use of PROs in clinical practice, Self-management recommendations for patients based on PROs) subsequently presented in the online survey. The online survey (open between 3 June and 19 July 2020) was completed by 233 HCPs from 33 countries. The highest preference was indicated for content on interpretation of PRO data (97%), clinical benefits of assessing PRO data (95.3%) and implementation of routine PRO data assessment (94.8%). Regarding learning methods, participants indicated a high preference for practical examples that use a mixed approach of presentation (written, audio, video and interactive).

**Conclusion:**

Educational needs for an integration of PROs in communication in clinical care and coherent implementation strategies became evident. These results inform the development of an e-learning course to support HCPs in the clinical use of EORTC PRO measures.

**Supplementary Information:**

The online version contains supplementary material available at 10.1007/s00520-021-06676-x.

## Introduction

Patient-reported outcomes (PROs) provide important supplementary information on patients’ subjective health status and symptom burden. A PRO, such as health-related quality of life (HRQOL), is a measurement of any aspect of a patient’s health status that comes directly from the patient without the interpretation of the patient’s responses by a physician or anyone else [1]. Evidence shows the value of integrating PROs into clinical practice to optimise symptom management, supportive therapy and patient-centred care and prolonged survival during oncological treatment [2–6]. Broad implementation into clinical routine, however, still remains a challenge, partly because of logistic and financial reasons as well as health care professionals’ (HCPs) lack of familiarity with the concept [7].

To support implementation of PROMs into clinical practice, educational tools for HCPs are needed which include information about PROMs but also take the clinical realities of limited human resources, time and funding into account. Digital training tools, such as web-based e-learning courses, proved to be feasible alternatives to onsite training [8–10]. Though recommended for successful integration of PROMs into clinical practice [11], there is a gap in targeted, validated and multilingual education on this topic [12–15]. We aim to develop an e-learning course to support HCPs in the routine use of PRO assessments in clinical oncology practice, using the example of EORTC PRO instruments. The EORTC PRO measurement system comprises the EORTC QLQ-C30, which is a core questionnaire to assess HRQOL in cancer patients and can be supplemented with disease-specific modules. To maximise levels of acceptance, a participatory approach is recommended during each stage of the developmental process, also regarding specific learning needs and training preferences [16].

This methodology goes in line with the recommended “contextual inquiries” of the holistic user-centred framework for the development of eHealth technologies, the “CeHRes Roadmap” [16–18]. Correspondingly, this study aimed to (1) explore the learning needs of oncological health care providers (with regard to course content and key issues on the implementation and use of PROMs in clinical practice) and (2) explore their preferences regarding e-learning methodology.

## Methods

A mixed methods approach was applied in an exploratory sequential design. A scoping literature review (in preparation for publication) regarding available e-learning courses on PROMs in oncology informed the development of the qualitative interview guideline. Confirmation of the predefined framework on learning content was supported by a subsequent international anonymous online survey. Complementary to the ethical approval of the leading study centre, each participating centre adhered to the local requirements and obtained ethical approval if necessary.

### Qualitative semi-structured interviews

Based on the results of the scoping review, a guideline for semi-structured interviews was developed (see supplementary material). In addition to open-ended questions, participants were asked to rate the relevance of predefined topics (i.e. concept/definition of PRO, available PRO measurement tools, choice of PRO measurement tools, timing and frequency of PRO assessments, choice of PRO measurement tools, interpretation of PRO scores, predefined examples of how to react to PRO data in clinical use and implementation issues) to be included in the course. Rating was performed on a Likert scale, from 1 (not at all relevant) to 4 (very much relevant).

#### Procedure

Participants (including patient representatives, physicians, nurses, allied health professionals and IT specialists) were recruited via the professional networks of the collaborators, applying a purposeful critical case sampling method [19]. The interviews were conducted either face-to-face, online or via phone by the participating study collaborators. Depending on the interviewee’s consent, the interviews were recorded and transcribed verbatim, or the interviewer took field notes during the interview.

#### Data analysis

All participants received a centre-related pseudonym to protect their identity. To optimise data protection, the initial pseudonyms were recoded by the data management centre across the centres into consecutive IDs. The interview transcripts were analysed by a multi-professional research team, including physicians, psycho-oncologists and PRO researchers, applying an inductive-deductive content analysis following the approach outlined by Mayring [20]. According to the content elements of the interview guideline, an initial codebook was constructed to which quotations were deductively assigned. Content not suitable for any existing category was subsumed into inductively created new categories. Transcriptions were independently analysed and discussed by four researchers (HS, SR, LW, MS) until consensus was reached. Themes and subthemes were reviewed and refined until researchers agreed that these reflected the essence of the complete dataset. Supplementary material Table 2 provides quotes from participants to illustrate the categories and themes (Supplementary material Table 2 is available as supplementary material). The results of qualitative data analyses informed the design of the subsequent online survey.

## International online survey

The international anonymous online survey was performed in order to confirm the relevance and complement the results of the interviews with a larger sample of the target group in various settings and countries. In addition, the survey aimed to assess educational needs and preferences regarding methods of presentation, to ensure generalisability of the e-learning course and applicability in different countries.

### Sample

The invitation to participate in the online survey was included in online newsletters/mailings from national and international professional associations (see “Acknowledgements”) and disseminated by different sources of the EORTC (i.e. EORTC Quality of Life Department, EORTC Events Office via EORTC website and EORTC Headquarter social media accounts).

### Procedure

The anonymous online survey was set up via Lime Survey (http://www.limesurvey.org/). Participants were informed about the procedures of data collection, storage and protection. According to the results of the semi-structured interviews (part A), eight major topics for a possible modular structure of the e-learning course were presented in the online survey. Participants were asked to rate the relevance of the listed topics on a Likert scale (not – a little – quite – very relevant) and to choose preferred methods of presentation for each topic from the provided options (e.g. visual, written, auditory). In addition, an open section for further comments and recommendations was provided.

### Data analysis

For the quantitative analyses, descriptive statistics (e.g. number and percentage) were applied via IBM SPSS Statistics 26. Qualitative data gained from comments in the open section were categorised according to the interview categories.

## Data integration

Final data integration of qualitative and quantitative data was conducted after completion of the online survey*,* for which a joint display was used [21].

## Results

### Sample description of the semi-structured interviews

Between December 2019 and May 2020, 73 semi-structured interviews were conducted with participants from Austria (*n* = 20), The Netherlands (*n* = 21), Germany (*n* = 19), United Kingdom (UK; *n* = 7) and France, Belgium, Norway, Denmark, Israel and Malaysia (*n* = 1 each), including physicians (*n* = 27), nurses (*n* = 21), psycho-oncologists (*n* = 10), patient representatives (*n* = 7), IT specialists (*n* = 4) and other professions (*n* = 4). Four participants (6%) were < 30 years of age, 39 participants (53%) were between 30 and 50 years of age, and 30 (41%) were older than 50 years. Overall, 16.4% (12/73) of all participants indicated not having any kind of prior experience with PROs.

### Sample description of the online survey

The international anonymous online survey was open between June 3 and July 19, 2020. The survey was completed by 233 HCPs from 33 countries, mainly aged between 30 and 50 years (62.2%) and 52% female. The sample represents the intended target group of HCPs, of whom 28.3% (66/233) indicated not having prior experience with PRO assessments in clinical practice (see Table 1).

**Table 1:** Sociodemographic characteristics of participants in (a) semi-structured interviews (*n* = 73) and (b) online survey (*n* = 233)

Variable		Interviews	Online survey
		*n* (%)	*n* (%)
Sex	Male	29 (39.7)	111 (47.6)
	Female	44 (60.3)	122 (52.4)
Age group	< 30 years	4 (5.5)	3 (1.3)
	30–50 years	39 (53.4)	145 (62.2)
	> 50 years	30 (41.1)	85 (36.5)
Country	Germany	19 (26.0)	36 (15.5)
	The Netherlands	21 (28.8)	29 (12.4)
	Italy		23 (9.9)
	Austria	20 (27.4)	20 (8.6)
	UK	7 (9.6)	18 (7.7)
	Belgium	1 (1.4)	14 (6.0)
	Sweden	-	12 (5.2)
	Japan	-	10 (4.3)
	France	1 (1.4)	8 (3.4)
	Spain	-	8 (3.4)
	Iraq	-	8 (3.4)
	Poland	-	5 (2.1)
	Ireland	-	5 (2.1)
	Portugal	-	5 (2.1)
	Denmark	-	4 (1.7)
	Switzerland	-	4 (1.7)
	Turkey	-	3 (1.3)
	Australia, Greece, India, Canada, USA	-	2 (0.9) (per country)
	Norway, Malaysia, Denmark, Israel	1 (1.4) (per country)	-
	Brazil, Finland, Canary Islands, Croatia, Lithuania, Norway, Philippines, Slovenia, Czech Republic, Cyprus, Egypt	-	1 (0.4) (per country)
Professional education (multiple answers possible)	Medicine	27 (37.0)	155 (66.9)
	Nursing	21 (28.8)	41 (17.6)
	Psycho-oncology	10 (13.7)	25 (10.7)
	Technical support assistant (e.g. medical, radiology)	-	4 (1.7)
	Patient representatives	7 (9.6)	-
	IT	4 (5.5)	-
	Physiotherapy (including sport science)	-	5 (2.1)
	Occupational therapy	-	1 (0.4)
	Social service (including social science)	-	2 (0.9)
	Other	4 (5.5)	
Previous experiences with PROs (multiple answers possible^1^)		*n* (%)	*n* (%)
None		12 (16.4)	66 (28.3)
Research		46 (63.0)	130 (55.8)
	< 2 years	3 (4.1)	2 (0.9)
	2–5 years	16 (21.9)	39 (16.7)
	6–10 years	6 (8.2)	36 (15.5)
	11–20 years	18 (24.7)	34 (14.6)
	> 20 years	3 (4.1)	19 (8.2)
Clinical practice		35 (47.9)	101 (43.3)
	< 2 years	4 (5.5)	8 (3.4)
	2–5 years	11(15.1)	24 (10.3)
	6–10 years	7(9.6)	15 (6.4)
	11–20 years	13 (17.8)	25 (10.7)
	> 20 years	0 (0)	29 (12.4)
Research and practice		25 (34.2)	102 (43.8)

^1^*n* = 38 did not indicate experience with PROs in years

### Merged data analysis and data integration

Qualitative analysis of the data assessed in the semi-structured interviews resulted in four main categories (A–D in Supplementary material Table 2) and ten subcategories (1–10 in Supplementary material file 2). A summary of categories and subcategories with representative quotes is presented in Supplementary material file 2. In the interviews, the embedded relevance ratings of the predefined themes showed highest ratings for basic knowledge on PROs (concept and definition) and self-management advice (95%), how to communicate (89%) and interpretation of PRO data and timing and frequency of assessments (88%). The categories were merged into content clusters representing eight topic areas that were subsequently presented in the online survey. The analysis of the online survey confirmed the results of the interviews with respect to the suggested topics and methods of presentation (see additional quotes in Supplementary material file 2). The relevance of all 8 categories listed was rated > 90%, with interpretation of PRO data (226/233, 97.0%), clinical benefits of assessing PRO data (222/233, 95.3%), implementation of routine PRO data assessment (221/233, 94.8%) and the use of PROMs and PRO data in clinical practice (220/233, 94.4%) receiving the highest relevance ratings (see Table 2). These were followed closely by basic information on PROs in clinical routine (219/233, 94%).

**Table 2:** Relevance and preference ratings of content and methods in the semi-structured interviews and the online survey

Content					Methods		
According to the semi-structured interviews (*n*** = 73)**		According to the online survey (*n*** = 233)**					
Topic as listed in interview guideline	Relevance^1^: 2–3 *n* (%)	Topic according to qualitative data analysis	Relevance^1^ assessed in online survey *n* (%)		Method of presentation *n*** (%)**^2^	Level of preference of method n (%)	
			0–1	2–3		0–1	2–3
Concept/definition 69 (95%)		1. Background, basic information on PROs in clinical routine (e.g. definitions of the concept of PROs)	14 (6.0)	219 (94.0)	Information, written	41 (17.6)	192(82.4)
Available PROM tools 57 (78%)					Information, spoken (e.g. podcast)	82 (35.2)	151 (64.8)
					Graphic presentations (e.g. pictures, flowcharts)	29 (12.4)	204 (87.6)
					Recommended literature (e.g. references/links to relevant publications)	72 (30.9)	161 (69.1)
		2**. **Benefits of PRO assessments in clinical practice	11 (4.7)	222 (95.3)	Information, written	40 (17.2)	193 (82.8)
					Information, spoken	80 (34.3)	153 (65.7)
					Graphic presentations	27 (11.6)	206 (88.4)
					Expert opinions (video clips)	88 (37.8)	144 (61,8)
					Experiences of patients (video clips)	60 (25.8)	173 (74.2)
					Recommended literature	74 (31.8)	159 (68.2)
Implementation 48 (66%)		3. Implementation of PRO assessments in clinical routine (e.g. planning and organization of the implementation process also including stakeholders, responsibilities, workflow)	12 (5.2)	221 (94.8)	Information, written	44 (18.9)	189 (81.1)
					Information, spoken	93 (39.9)	140 (60.1)
					Graphic presentations	31 (13.3)	202 (86.7)
					Expert opinions	84 (36.1)	149 (63.9)
					Examples of integration of PROs into clinical workflow (written, audio-visual)	19 (8.2)	214 (91.8)
					Examples of successful implementations in different clinical settings (written, audio-visual)	22 (9.4)	211 (90.6)
					Recommended literature	73 (31.3)	160 (68.7)
Choice of PROM tools 53 (73%)		4. Setup of PRO assessments for clinical application (e.g. instruments, assessment times and frequencies)	14 (6.0)	219 (94.0)	Information, written	46 (19.7)	187 (80.3)
Timing and frequency 64 (88%)					Information, spoken	97 (41.6)	136 (58.4)
					Graphic presentations	28 (12.0)	205 (88.0)
					Examples of the setup of PRO assessments in different clinical settings (written, audio-visual, interactive)	19 (8.2)	214 (91.8)
					Expert opinions	87 (37.3)	146 (62.7)
					Recommended literature	80 (34.3)	153 (65.7)
Interpretation of PRO 64 (88%)		5. Interpretation of PRO data (e.g. general understanding of how to interpret the PRO scores of patients, normative values, thresholds for clinical relevance, minimal important differences)	7 (3.0)	226 (97.0)	Information, written	31 (13.3)	202 (86.7)
					Information, spoken	94 (40.3)	139 (59.7)
					Graphic presentations	16 (6.9)	217 (93.1)
					Recommended literature	58 (24.9)	175 (75.1)
How to communicate 65 (89%)		6. Integration of PROs into the communication with patients (e.g. ward rounds, consultations, challenging situations, high symptom burden, distress)	19 (8.2)	214 (91.8)	Information, written	51 (21.9)	182 (78.1)
Probing questions 59 (81%)					Information, spoken	86 (36.9)	147 (63.1)
					Graphic presentations	42 (18.0)	191 (82.0)
					Theoretical information on how to integrate PROs into the communication with patients	48 (20.6)	185 (79.4)
					Video based examples for communication based on PROs	51 (21.9)	182 (78.1)
					Interactive case vignettes (e.g. different diagnoses, stages)	44 (18.9)	189 (81.1)
					Expert opinions	98 (42.1)	135 (57,9)
					Experiences of patients	54 (23.2)	179 (76.8)
					Recommended literature	90 (38.6)	143 (61.4)
Supportive measures 53 (73%)		7. Use of PROs in clinical practice (e.g. reaction to results, initiation of clinical action, involvement of psycho-oncology)	13 (5.6)	220 (94.4)	Information, written	51 (21.9)	182 (78.1)
Further diagnostics 53 (73%)					Information, spoken	98 (42.1)	135 (57.9)
					Graphic presentations	39 (16.7)	194 (83.3)
					Expert opinions	77 (33.0)	156 (67.0)
					Experiences of patients	57 (24.5)	176 (75.5)
					Examples of clinical consequences based on PRO results (written, audio-visual, interactive)	21 (9.0)	212 (91.0)
					Recommended literature	84 (36.1)	149 (63,9)
Self-management advice 61 (95%)		8. Self-management recommendations for patients based on PROs (e. g. for various reported symptoms like fatigue, sleep disturbance, pain, gastrointestinal symptoms)	23 (9.9)	210 (90.1)	Information, written	43 (18.5)	190 (81.5)
					Information, spoken	87 (37.3)	146 (62.7)
					Graphic presentations	55 (23.6)	178 (76.4)
					Expert opinions	82 (35.2)	151(64.8)
					Experiences of patients with self-management recommendations (video clips)	43 (18.5)	190 (81.5)
					Examples of self-management recommendations (written, audio-visual, interactive)	28 (12.0)	205 (88.0)
					Recommended literature	89 (38.2)	144 (61.8)

^1^1, not at all; 2, a little bit; 3, quite a bit; 4, very much

^2^Multiple answers were possible

**Fig. 1:**
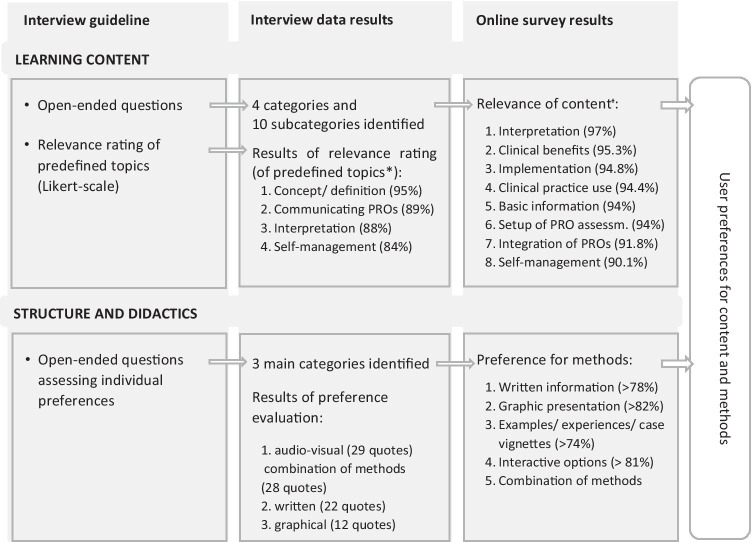
Joint display of qualitative and quantitative data

Qualitative and quantitative findings of both methods are presented in a joint display (see Fig. 1). Table 2 provides a detailed summary on participants’ ratings of relevance of suggested topics for the content and the methods of the e-learning course from both the semi-structured interviews and the online survey. On these findings, we based the final selection of topics for the learning content and structure and didactics of the e-learning programme, including methods of presentation (see Table 3).

**Table 3:** Final selection of content and methods

	Topic	Methods
1	Basic information on PROs in clinical routine	Written information, graphics
2	Benefits of PRO assessments in clinical practice	Written information, graphics
3	Implementation of PRO assessments in clinical routine (including facilitators and barriers)	Written information, graphics Examples of integration of PROs into clinical workflow Examples of successful implementations in different clinical settings (and pitfalls)
4	Setup of PRO assessments for clinical application	Written information, graphics Examples of the setup of PRO assessments in different clinical settings (and pitfalls)
5	Interpretation of PRO data	Written information, graphics
6	Integration of PROs into the communication with patients	Written information, graphics Interactive case vignettes
7	Use of PROs in clinical practice (i.e. PRO-based actions)	Written information, graphics Examples of clinical action and pathways based on PRO results
8	Self-management recommendations for patients based on PROs	Written information, graphics Examples of self-management recommendations (and patients using them: video clips)
General and structural aspects		Interactive components to enhance knowledge-practice transfer regarding the use of PROs in clinical practice
		blended learning to be considered, depending on structural possibilities
		Certification/accreditation points (if feasible), test of learning progress

#### Learning content

In the following, each topic section is presented in short. For representative quotes on each section, please refer to Supplementary material file 2.

1. **Basic information on PROs in clinical routine**
  Theoretical knowledge and basic information regarding the concept of PROs, measurement tools and assessment modalities was regarded important in order to reach and inform HCPs, especially those who are new to the field. This includes pointing towards the underlying biopsychosocial model and the importance of including the patient perspective into symptom management for a comprehensive treatment approach.
2. **Benefits of PRO assessments in clinical practice**
  Building on basic information, evidence regarding the potential benefits of PRO assessments for clinical practice should be presented and explained to increase motivation to use PROs in clinical practice. Their contribution to a potential survival benefit was judged to be most important to convince oncologists. Additionally, evidence regarding the benefit of PROs for a comprehensive, more structured symptom assessment should be included, even more so in patients reluctant to report their symptoms. Other mentioned areas of benefit are enhanced symptom management, PROs as additional information for treatment planning and facilitation of patient-HCP communication as well as a more active involvement of the patients in decision-making.
3. **Implementation of PRO assessments in clinical routine**
  Participants underlined the importance of addressing the implementation process regarding institutional and individual aspects on different organisational levels (i.e. HCPs with decision-making capacity or in management positions, individual and team perspectives). Implementation issues further included general planning of the implementation of PRO assessments, including costs, resources, stakeholders and technical issues. A stepwise approach for implementation, including identification of and collaboration with motivated colleagues and onsite coaching, was suggested. Awareness of individual and organisational barriers and facilitators (e.g. individual barriers for staff, patient ability and motivation) was described as important. The supporting role of motivated colleagues and senior peers acting as leaders of integration was highlighted.
4. **Setup of PRO assessments for clinical application**
  Considering the setup of PRO assessments, the choice of instruments, timing and frequency (differing with regard to type of disease and treatment phase), emphasising the necessity to keep administrative burden as low as possible was rated to be relevant. The course should also comprise information on available options for electronic data assessment and integration of PRO systems into medical records. Further important aspects to consider when setting up routine PRO assessments include, among others, operative responsibilities, workflow and changes in workflow, inter-professional collaboration including engaging staff in the initial phases of implementation as well as building realistic expectations concerning the time needed to invest.
5. **Interpretation of PRO data**
  Regarding practical aspects of PROs like treatment planning and symptom management, participants emphasised that the e-learning course should comprise information on the interpretation of PRO data including a general understanding of how to interpret the PRO scores of patients, normative values, thresholds for clinical relevance and minimal important differences.
6. **Integration of PROs into communication with patients**
  Increased attention should be set on the topic of communicating with patients about PROs on ward rounds, in consultations or in challenging situations such as high symptom burden or distress. Likewise, more specifics on how to proceed with further diagnostic questions on reported symptoms and impairments should also be included in the training.
7. **Use of PROs in clinical practice**
  Participants stated the need for recommendations on PRO-based clinical actions for patient-reported symptoms, aiming to make data usable in clinical practice. This may include supportive care options available for specific symptoms or problems such as sexuality and fatigue. Especially nurses emphasised the need for a description of concrete procedures that need to be followed when PRO data warrant clinical action. Also, the consideration of ethical and legal aspects of PRO data assessment in clinical practice should be addressed.
8. **Self-management recommendations for patients based on PROs**

While providing self-management recommendations for various symptoms (e.g. fatigue, sleep disturbances, pain, gastrointestinal symptoms) was reported to be relevant especially in the out-patient setting, the necessity of conveying theoretical understanding of self-management education was also addressed. This includes refining the capacity of HCPs to differentiate between available recommendations and materials in this area to support them in choosing the most suitable tools for their patients.

#### Structure and didactics of the course

##### Structure of the e-learning course

Participants shared the opinion of the need for tailoring course levels to various learning needs, supporting a modular structure for the e-learning course. The importance of usability, including navigation within the programme and easy access, was underlined. Despite the obvious possibilities and advantages of e-learning, awareness of the limitations of this approach, including individual or structural barriers for its use, was deemed important.

Relating to the time frame, interview participants indicated their acceptance for 10–25 min per unit and 1.5–2 h for the whole programme. Moreover, 52.1% opted for receiving a certificate if possible or to only receive a certificate when accreditation is provided.

##### Presentation of learning content

With regard to presentation formats, interview data suggested high preference for a combination of methods, such as audio-visual and written information. Correspondingly, in the online survey participants indicated preference for written information (> 78% in all content categories) and graphical presentation (> 82% in all content categories, except “self-management”). Participants would highly appreciate a presentation of practical examples of integration of PROs into the clinical workflow (92%), clinical actions based on PRO data (91%), successful implementations in different clinical settings (90.6%) and self-management recommendations (88%) as well as case vignettes about patients with different diagnoses (81.1%). Qualitative comments suggested providing successful best practice examples showing the advantages, feasibility and acceptance of PROs in clinical practice presented by respected peers. Examples of unsuccessful implementations to increase awareness of potential problems and pitfalls were mentioned as well. Additional suggestions for methods of presentation in the online survey included screencasts, webinars and videos.

##### Interactive components

Participants stressed that the course should include interactive components (e.g. regarding the case vignettes, interpretation of data, communication) to facilitate analysing the content and creating individual relevance and enhance knowledge-practice transfer supplemented with options for personal exchange between users and the possibility to combine the e-learning course with face-to face training. Participants would also appreciate options for assessing individual learning progress (e.g. quizz).

## Discussion

This study reports on the first step in developing an e-learning course on the use of EORTC PRO measures for routine PRO assessment targeting oncological health care providers using a user-centred design methodology. Herein, we present the results of the analyses of stakeholder perspectives on learning content and methods.

### Preferences for learning content

With respect to topics that need to be addressed in the e-learning course, HCPs indicated the highest relevance for the interpretation of PRO data, clinical benefits of assessing PRO data, implementation of routine PRO data assessment and the use of PROMs and PRO data in clinical practice. The wish for more standardisation of procedures and increasing the sense of ownership of and the involvement in the process setup was expressed. These results are in line with recent findings on HCPs’ perspectives on implementing PROs in routine clinical care in oncology [22–24]. HCPs included in our study recognise the potential to promote earlier intervention and more holistic approaches to oncology care but were concerned about cases when they might feel unable to interpret and adequately respond to the issues identified (e.g. if feasible solutions were unavailable). The need to educate providers about the benefits and value of PROs beyond current clinical approaches and to develop coherent implementation strategies also became apparent [22, 23]. Further implementation aspects raised by participants of our study correspond to key issues reported by Snyder, e.g. goals of assessments, selection of questionnaires, timing and mode of assessments, interpretation of the scores and clinical consequences [24].

The general question of how to motivate other colleagues and decision-makers to opt for routine PRO assessment emerged from most interviews and does generate different answers depending on the respective HCP target group. While a possible survival benefit was reported to be especially motivating for oncologists, standardised care pathways were indicated to support the engagement of nursing professionals. Critical comments emphasised the necessity to address the fundamental question of how PROs, and thus the patient perspective, can be integrated into clinical care in a way that is “actionable” for routine clinical settings. Attitudes towards PROs are often tainted by the fear of losing the human connection with the patient as the centre of physician–patient-interaction, often referred to as empathy [25].

Both the literature and our study show that the following three premises have still not reached the collective knowledge of HCPs, at least in this sample: (1) the patient’s perspective is valid, (2) the patient is an important source of subjective symptom information that is otherwise not available, and (3) empathy is an essential aspect in the encounter between people (and therefore also in the clinical encounter). However, a structured measurement of symptom burden will still supplement symptom assessment in the medical interview and allow monitoring over time. This aligns with Stephen [12], stating that when approaching the development of (nursing) curricula, person-centred care should be embedded within several courses and incorporated across the curriculum. According to the latter, areas of interest should include knowledge on how to facilitate PRO assessments, how to implement care based on the knowledge gained through PRO data, how to evaluate care based on the feedback given according to PRO data as well as competence in administering PRO instruments, reviewing the following assessments and care planning. Likewise, this should include a balanced presentation of organisational requirements for the adaptation of an effective PRO-based assistance and benefits of the latter for patient outcomes.

### Preferences for structure and didactics of the e-learning course

Considering a digital learning format, HCPs expressed the importance of examples of integration of PROs into the clinical workflow, clinical actions based on PRO data, successful implementations in different clinical settings and self-management recommendations as well as case vignettes about patients with different diagnoses. Integration of examples of successful *best practice* was suggested but also those of unsuccessful implementations to increase awareness of potential problems and pitfalls. Interactive components such as video examples of simulated consultations with oncologists using PRO data have been previously reported as desired learning formats [11, 13, 18].

The preferences for a modular approach and tailoring course levels to learning needs were also stated in the context of other digital learning tools where HCPs expressed the importance of a simple and visually attractive e-learning environment with a combination of (evidence-based) theory, modelling videos and illustrations [18]. Different HCP target groups with differing clinical obligations, service settings and associated fields of interest might require different content foci, e.g. general planning and organisation of the implementation process, including awareness of structural barriers and limited resources, might be more interesting for HCPs who are in a position to decide on and plan implementations.

### Strengths and limitations

Strengths of the project are the stepwise design comprising a scoping review of the relevant literature, qualitative interviews and the online survey resulting in quantitative data, corresponding to the “CeHRes Roadmap” for the development of eHealth technologies [16–18]. The high participation rate enabled a rich qualitative and quantitative database. The purposeful sampling provided perspectives of professionals of various professional backgrounds as recommended [16]. The international sample of the online survey allowed a more detailed insight and interpretation of the results for the intended international target population, differing in their level of familiarity with and expertise in assessment and interpretation of PROs and different health care systems.

## Conclusion

Educational needs regarding knowledge of and competences in the integration of PROs in communication and clinical care became evident, implying possible different foci of learning content and topics of interest across levels of clinical engagement. Data imply HCPs’ wish for more standardisation of clinical procedures of PRO assessment and initiation of PRO-based clinical pathways. The importance of concrete examples of integration of PRO systems and clinical use based on PRO data was highlighted. Regarding e-learning methodology, the modular approach and interactive case-based examples were considered important features.

## Supplementary Information

Below is the link to the electronic supplementary material.

Supplementary file1 (DOCX 22 KB)

Supplementary file2 (DOCX 42.9 KB)

## Data Availability

Data is available upon request.
