# Supplementary material for: Developing an e-learning course on the use of PRO measures in oncological practice: health care professionals’ preferences for learning content and methods
Source: Support Care Cancer. 2021 Nov 19;30(3):2555–67. doi: 10.1007/s00520-021-06676-x (PMC8794964; doi:10.1007/s00520-021-06676-x)
Supplement: Supplementary file 1 — Supplementary file1 (DOCX 22 KB) [file 520_2021_6676_MOESM1_ESM.docx]

1. **LEARNING CONTENT**

**1.a** What would you like to learn about routine PRO assessments?

**1.b.** What should a colleague new to the field of PROs learn about routine PRO assessments?

**1.c.** Following, I will name possible content and would like you to rate the aspects for relevance on a scale from 1 (not at all relevant) to 4 (very much relevant).

**Theoretical knowledge**

1. Concept/ definition of PRO
2. Available PRO measurement tools
3. Choice of PRO measurement tools
4. Timing and frequency of PRO assessments

**Clinical use**

1. Choice of PRO measurement tools
2. Timing and frequency of PRO assessments
3. Interpretation of PRO scores
4. Examples of how to react to PROs
   - examples of communication with patients about the results incl. probing questions to further differentiate and explore individual patient experience
   - examples of supportive measures and possible care concepts
   - examples of further diagnostics triggered by PROs incl. communication with other HCP
   - self-management advice for patients based on individual PROs

**Implementation issues**

e.g. how to implementing PRO assessments in clinical routine (who has to be involved, what barriers and facilitators should be considered)

Please add any aspect you think is missing from this list.

1. **IMPLEMENTATION OF THE USE OF PRO ASSESSMENTS IN CLINICAL ROUTINE:**

**2.a**. Can you think of any aspects hindering the use of PROs in your clinical practice?

**2.b.** Which aspects could facilitate the use of PROs in your clinical practice?

**2.c.** Who do you think should be involved when planning implementation of PROs in clinical routine?

**2.d.** What else should be considered when planning implementation of PROs in clinical routine?

1. **LEARNING METHODS**

**3.a.** Which methods do you consider would be useful in an e-learning programme about PROs?

**3.b.** From this list of methods on how the content of an e-learning programme could be presented: Do you have preferences for some methods listed above? Which are your personal top three?

1. written
2. audio (e.g. podcast)
3. video tutorials
4. infographics
5. mixed modes of presenting information (written, audio, video)
6. case presentations (written, with pictures)
7. case presentations (video)

**3.c.** How do you think that colleagues who are not open for the potential benefit of the use of PRO assessments in clinical routine could become interested in learning something about PROs?

1. **Additional aspects:** Is there anything else you would like to add?
