# Supplementary material for: Developing an e-learning course on the use of PRO measures in oncological practice: health care professionals’ preferences for learning content and methods
Source: Support Care Cancer. 2021 Nov 19;30(3):2555–67. doi: 10.1007/s00520-021-06676-x (PMC8794964; doi:10.1007/s00520-021-06676-x)
Supplement: Supplementary file 2 — Supplementary file2 (DOCX 42.9 KB) [file 520_2021_6676_MOESM2_ESM.docx]

Supplementary Table 2. Main categories (A-D) and subcategories of qualitative data with representative quotes from interviews (ID) and online survey (OS) regarding I. content and II.

|  | **MAIN CATEGORIES** | **SUB-CATEGORIES DERIVED** | **Representative Quotes** |
| --- | --- | --- | --- |
| 1. **CONTENT** | 1. **Basic information** **on PROs in clinical routine (i.e. definition and information on concept of HRQOL and PROs)** | | |
|  |  |  | - “*It's very important that there is a definition of PROs for all (...) That they at least have the basic knowledge about what is entails.”* (ID 25) - “*I would phrase it as simply as possible: what it involves, how it is defined and why we do it* [PRO assessment].” (ID15) - *“[…]. It is not so much about PROs, but about the understanding that the patient perspective is extremely relevant. Treatment without this patient view is now completely inadequate. That's actually the point at which it [education] fails. That we start the education in the middle and that this basic understanding is often missing."* (ID51) - “*I think it's really important to understand the concept that it's something that comes from the patients and that we cannot quantify or judge the information*.” (ID24) |
|  | 1. **Benefits of PRO assessments in clinical practice** | | |
|  |  | Including information on:   - Evidence on benefits for clinical practice e.g. positive effects on symptom management, patient-HCP communication, survival etc.) - Holistic approach, comprehensive symptom assessment - Additional information for treatment planning - Active involvement of patients in decision-making and treatment planning | - *“In oncology, its survival, which is the main outcome. So start showing the survival benefit [of PRO assessment]. That will be quite useful to persuade oncologist.”* (ID28). - *“That nothing is overseen* *especially for shy and reserved patients who otherwise would not communicate their symptoms. In these cases PROs might help.”* (ID18) - *“It [PRO assessments] could be a benefit because if you gather symptoms more structured, the symptom management could be optimized.”* (ID12) - “*It* [PRO assessments] *may help to better prepare the consultations and structure conversations*.” (ID73) - “*And it is highly appreciated by the patients. Because you bring up the things that seem to matter to them*.” (ID55) - *“The opinion of patients can be used to guide the physician in the treatment. The patient is the centre of care! So, look at what we don't know about the disease from another point of view.”* (OS) |
|  | 1. **Implementation of PRO assessments in clinical routine** | | |
|  |  | 1. **General planning and organization of the implementation process including awareness for organisational / structural facilitators and barriers**     - general policy and guideline (e.g. value based health care)    - stakeholders to be included    - necessary resources (e.g. technical aspects, costs, staff)    - scope and time frame of implementation (clinic, departments etc.)    - general responsibilities    - data protection issues, ethical aspects    - training of staff | - *“When you want to implement PRO assessment, you need to consider political aspects (how the organisation works together, how the different professions work together).”* (ID 60) - *“It's a whole clinical issue, I guess. This needs to be leadership from the top down, if you want to implement this you need support from [whatever is involved] institution or hospital.”* (ID 20) - “*I think what's also beneficial is how the basic attitude of the clinic is - is the institution behind the initiative?* *You need the senior management on board. So that are the people who provide the money On the ground, you got to engage the whole team. (...) If people are coming to the clinics, you've got to engage the person who is at reception meeting the patients. (...) You need to engage nurses as well, and then you go to the physician or specialist nurses.”* (ID12) - “*General acceptance - some of the seniors within the department have to promote this*.” (ID25) - *“As a university hospital, it is of course an insanely large structure in which a wide variety of specialised departments must be involved. In this case, the people responsible for data management and data protection, the people responsible for the technological infrastructure, the doctors and the nursing staff on the respective ward have to be taken into account, the clinic management, the nursing management of the clinic (...) of course, the patients (...) self-help groups.”* (ID 05) - *“The nurses, in a hospital, nothing will work without nurses. Not only the doctors but also a nurse should be there who is responsible (...) The primary contact, first of all, is always with the nurse.”* (ID13) - *“Maintenance should not be underestimated, and by that I mean least of all the technical area.”* (ID55) - *“Which departments within the clinic can be covered or is it something for the whole clinic - is that predefined or are there any gaps?”* (ID22) - *“I think the employees just need to be trained in this and not everyone will be ready.”* (ID48) - *“The biggest barriers are all kinds of staff turnover - the less permanent staff you have and the more staff turnovers, the harder it gets to make sure that everybody is well informed about the process.”* (ID 15) - *“In the routine, what is tedious is that you constantly have to instruct new people, because the staff fluctuation in the outpatient clinic is naturally.”* (ID55) - *“So the first thing I would do is explain it to the nursing management in the outpatient department […] and then they [the nurses] have to be trained.]”* (ID 01) - *„For the groups of people who introduce the patients to the assessment, coaching is of course certainly good. And vice versa for those who get the result in their hands, i.e. the doctors, it is good to be told what to do with it, how to integrate it into your workflow.”* (ID55) - *“Again, the implementation procedure must be described, otherwise the nursing staff cannot envision it. The more concrete the better.”* (OS) - *“Think about the integration into clinical routine involving all professional groups. Consider the implementation planning, otherwise the PROs will not be adopted.”* (OS) |
|  | 1. **CONTENT** | **2. Setup of PRO assessments for clinical application**   - choice of appropriate tools - timing and frequency - methods of data collection | - “*I think [in the e-learning course] it would need a good overview of the different PRO instruments, what possibilities there are, for which diagnosis and patient groups. Information on how to use them [PRO measures], how they can be more easily administered on the wards in practical use, without much additional effort*.” (ID43) - “*The time and frequency of detection is certainly different depending on the disease, whether I have to screen more often or whether I say it is sufficient if I screen at baseline, after three months, after 6 months - that certainly depends on the type of disease.”* (ID48) - *“They should have a bit of knowledge on what makes a good PRO for them to make a good choice for themselves for what they need it for. PROs are fit for purpose, and if you use it for a wrong purpose, you won't get anything out of it they need to be right for them.”* (ID29) |
|  |  | 1. **Specific planning**  - practical issues (e.g. structured procedures for the integration of specific assessments in clinical routine, operative responsibilities, workflow and changes in workflow) - instructions for HCP for the use in clinical routine and instructions for patients - inter-professional collaboration comprising also teamwork and trust | - *“Perhaps, the commitment of responsibilities and competencies: Whose job is it: to provide information and training to patients and to see that the questions are answered and perhaps also to prepare the data.”* (ID 03) - *“The procedure should be very simple and a kind of self-explanatory for them to start using it.”* (ID 28) - *“(There needs to be) Information on patient instructions... Basic information should be provided for the patients, why this is being done and what advantages it has, that it is in the patients' interest to fill it in. To do this one would have to instruct the practitioners so that they can instruct the patients.”* (ID 43) - *“That the benefit is there for both sides, not only for the nursing staff, if the information can be prepared and passed on. Both sides [physicians and nurses] profit from it [..].”* (ID48) |
|  |  | 1. **Awareness for individual barriers**  - lack of knowledge - also regarding doubts about and choice of appropriate tools - staff attitude and motivation, - willingness to change (established clinical routines, role & culture) | - *“[...] the aspects are actually the lack of understanding* [of what PROs are good for] *and this argument that comes up very quickly, is that it all takes too much time and we have no time and no money. I have the feeling that this is always the first reaction* [saying No] *and that it takes time*. *Someone has to be able to sit down and listen*.” (ID51). - *“There is a very general phenomenon. When something new comes along, no matter what it is, it makes some people fundamentally insecure. No matter what it is. "What is it? We've never done that before, we've always done it differently. I know my patients anyway, I know how they are." I think you always have such systemic resistance, whatever you do.”* (ID55) - *“PRO assessment in clinical practice is very relevant. The common practice of application in clinical practice of instruments developed for quantification of PRO in clinical trials is, however, inappropriate.”* (OS) |
|  | 1. **CONTENT** | 1. **Patient issues**: ability & motivation | - *“The communication level must be adapted to the patient. One should not overwhelm a patient with too many words he/ she may not be familiar with but should also address the educated patients at an appropriate level.”* (ID 18) - *“The questionnaire being too long [...] typeface too small.”* (ID 13) - *“And I think to patients for them to learn about PROs it really depends on the context. Some hospitals in their waiting rooms will have television playing. Perhaps there could play videos about PROs.”* (ID 20) - *“Sometimes this is one of the barriers that the patients refuse to participate because they didn't get the right idea of what we're doing.”* (ID 26) |
|  |  | 1. **Facilitators for the implementation process**: how to motivate e.g. personal support and onsite coaching, agents of change | - *“But maybe you can only reach a certain number of people, it's about getting those engaged people first. And then they're going to be the leaders.”* (ID 29) - *“You need a ‘champion’, and that could be the head of the department, could be another senior physician, could be a nurse*.” (ID 28). - *“And showing to the clinicians what this [implementing PROMs into clinical practice] would do for them: reduce pressure on clinics; improve patient compliance; improve outcomes - properties that fall back to the clinical team.”* (ID 21) - *“It should be made clear that the whole thing [PRO assessment] is not intended to replace the doctor's consultation, but rather to enrich it.”* (ID 18) - *If you find a way of integrating PROs so that they make life simpler for the whole team, then that will be beneficial. (...) I think at the moment, most of the implementation is about adding PROs to the existing system. - Physicians and nurses are concerned about doing ever more tasks and taking longer. I think one facilitator would be if you actually replace an existing service with PRO service. If you reorganize the services including PROs that will be facilitators* (ID 28) - *Regarding the time factor, it should be made clear that things take more time at first, but that in the long run, this can save time* (ID 18) |
|  | 1. **Use of PROs in clinical practice** | | |
|  |  | 1. **Interpretation of PRO data:**   knowledge on how to interpret the PRO scores of patients including normative values, thresholds for clinical relevance, minimally important differences | - *“[…] when collecting these data, you need to know how to interpret them afterwards.”* (ID 14) - *“Interpretation how to interpret the PROs – (…) so how could you interpret responses from an individual versus sample level.“* (ID 08) - *“Because you should have an understanding of how to classify an absolute score in the context of population or people with the same disease, e.g. if all have a score of 90, then 90 isn't much, it is normal.”* (ID 03) - *“People need to have that. A number is just a number. Oh, my score was ten, ten out of what, out of a hundred, out of a thousand? It is so important to get score interpretation and the meaning of change. So, what does a stand-alone score mean and what does a score change mean by ten, is that meaningful?”* (ID 29) - *“What is a clinically relevant difference, and relate this to medical interventions.”* (ID46) - *"Is there a cut-off value from which a measure must be taken immediately? What role does the nurse play in this? [...] - well, specific tasks of my professional group. Are there degrees of severity? Is it a diagnostic instrument or a screening instrument? When do we become active and what actions are derived from it?"* (ID 45) |
|  |  | 1. **Integrating PRO data into the communication with patients:**   explaining PRO assessments to patients, communicating PRO results to patients, further exploration and differentiating questions | - *“The program should be on the one hand helpful to reflect my way of dealing with patients and the consequences for my practical actions […] and on the other hand, it should be something like a guideline for conversations with patients [in the medical round or outpatient setting].”* (ID12) - *“How do I include it into the conversation, how do I deal with questions, how do I deal with the emotions that arise, the uncertainties*.” (ID51). - *“It is no different from any other communication training. Where if a person comes with a long list of symptoms. We say: Sorry, to hear, you had so many bad experiences? So, which is the most important one so that we can start from there. So, it's no different with PRO. You then say: You have many problems. Which one is the most important for you to start with?”* (ID28) - *“Use it as cues. You ask the patient. Then can be a cue for them to tell you something that they wouldn't have told you otherwise.”* (ID19) |
|  | 1. **CONTENT** | 1. **PRO-based clinical action**:   initiation of clinical action also including counselling, supportive therapy, pathways and clinical guidelines | - I think, having established supportive care pathways to respond to PRO issues, like physical function or fatigue difficulties, is important, for people to respond. So even when patients report [symptoms], clinicians may not necessarily know how to deal with this. So having established pathways for e.g. fatigue, severe fatigue etc. and what resources are available will give people the confidence to discuss symptoms when people bring them up on their PRO assessments (ID20) - *“When do we become active and what measures are derived from it?”* (ID45). - “[would like to learn] *supportive care options available to specific symptoms, especially related to problems as sexuality, fatigue. And not e.g. pain* (we know already what to do)” (ID40). |
|  |  | 1. **Self-management-recommendations for patients based on PROs:**   for various reported symptoms like fatigue, sleep disturbance, pain, gastrointestinal symptoms | - *“Especially in the outpatient setting, patients should know what they can do for themselves with easy to implement supportive measures.”* (ID14) - *“[...] if you give patients self-management measures, then this means something for the patient's own development”* (ID51). - That contributes to the benefits for patients. We have a clinical algorithm behind the PROs that you could generate advice for the patient, and we have a website where there is more detailed advice. And we know from the patients that the valued this. Mainly because a kind of reassures them that what they do is correct. And our advice is captured into mild and moderate problems that do not require medical attention immediately. - For high temperature severe pain or vomiting, they will get the advice to contact hospital NOW (ID28) - *“One or two examples of self-management recommendation for very common symptoms is relevant to clarify the entire process of using PROs in clinical care.”(OS)* |
| 1. **METHODS** | **Different forms of presentation** | | - *“If they meet someone they respect, some professor, some person who says: This is really something you ought to do. That is really what brings on the change. [...] A respected colleague telling about PRO benefits”* (ID25) - *I think video tutorials are very appealing because you can work with both video and audio. You can present exemplary situations and very concrete practical situations. For example: how the patient uses it; how the nurse reacts to it; how physician-patient-consultations might take place, how people react to it, and how the nursing process then works (ID22)* - *“Include the example on how to communicate that would be great. Because sometimes we never expect what the patient will say. Whenever there is an example at least, we can anticipate, and we can communicate to the patient more effectively* (ID27) |
|  | **Interactive components** | | - *“[...]thought provoking questions in between; include some interactive aspects” (IBK19)* - *“Interactive websites, try it yourself“ (ID46)* - *“[...] maybe something that is more interactive. You need to be part of the discussion. We have a lot of e-learning programmes in our medical centre and almost everything is interactive.”* (ID 26) |
|  | **Structure of the e-learning course** | | - *“To pick and choose bits and pieces that you want to learn about.”* (ID 28) - *“Not too long. The time expenditure should be manageable.”* (ID 63) - *“I think basic information on the concept of PROs is useful for people new to the field and/or trying to introduce PROs in their own hospital. For people that have experience with PROs this specific topic may not be useful in a course, as they probably know this. I think this kind of information during a course depends on your audience.”* (OS) |
